# On Dysentery

**Published:** 1853-07

**Authors:** W. W. Fritts

**Affiliations:** Carlisle, Ky.


### WESTERN JOURNAL

OF

### MEDICINE AND SURGERY.

---

JULY, 1853.

---

ART. I.—*On Dysentery.* An Inaugural Dissertation, submitted to the Trustees and Medical Faculty of the University of Louisville, February 1st, 1853, for the degree of Doctor of Medicine. By W. W. FRITTS, M. D., of Carlisle, Ky.

I propose giving a brief sketch of Dysentery, as it occurred in a portion of Nicholas county, Ky., in the latter part of the autumn of 1851.

The portion of country invaded by this disease, in an epidemic form, was so small, and its outlines so well defined, that I have thought it might not be out of place to describe very briefly the section thus visited, especially as it has heretofore been considered, if not the most healthy, amongst the healthiest portions of the county; and facts fully sustained this opinion until, in the year

1849, the cholera broke out in the lower part of this district, and in no other part of the county, proving fatal to quite a number of the inhabitants. The northeastern portion of the county is quite broken and pretty heavily timbered, except bordering on the water courses and valleys, which, in greater part, have been cleared out by the inhabitants, who dwell mostly in rude cabins situated on the low grounds. The soil, though not deep, is a rich black loam, resting upon clay, bedded at a depth of some six or eight feet, upon alternate layers of lime and soapstone, and is of that peculiar lively kind so well adapted to the growth of Indian corn and hemp; the former of which is the principal crop cultivated in this section. The water used for drinking and culinary purposes is limestone. The timber is white, red and black oak, poplar, ash, hickory and sugar-maple, with an under-growth of blackhaw, black-hickory, scrubby oak, &c., &c.

The water courses flowing in a north-easterly direction empty into the Licking river; they are small, but very numerous, passing over exceedingly rough limestone beds, having a fall of some seventy feet to the mile.

The dividing ridge separating these streams from those which flow in an almost opposite direction, emptying into the Slinkston fork of Licking and its tributaries, is the highest elevation of land on the line from Maysville to the city of Lexington, being five hundred and eleven feet above the Ohio at low water at the city of Maysville, and three hundred and eighty-four feet above the Licking at the same stage of water, which latter is but about five and a half miles from this summit. This north-eastern slope, extending from the highlands near Carlisle, the county seat of Nicholas, to Licking and some miles up and down said stream, is the district in which Dysentery appeared in an epidemic form. On one of the streams, I believe there was not a habitation where the disease did not prevail, and scarcely an individual who was not the subject of an attack of more or less violence. The disease, however, extended beyond this boundary, but its violence was always proportioned to the distance at which it occurred from the infected district, as was manifested by the fact that cases, as a general thing, y elded to, or reisisted remedies, just in proportion as they were removed from or near the centre of the infected region.

Having briefly described the district invaded by Dysentery, it remains for us to discover, if possible, a cause existing in the boundary specified to which we can plausibly attribute the prevalence of the epidemic; a cause existed most certainly—but what was that cause?

The following general and local facts are detailed because of the probable influence they had in the production of the disease under consideration.

The summer and autumn of 1851 were most remarkable for the extreme drought which prevailed throughout northern Kentucky. It was remarked by the oldest citizens, that a drought to the extent of the one which this season prevailed, had not occurred in the last fifty years. Streams and springs which had heretofore afforded an abundant supply of pure running water, were now dry, with the exception of here and there a stagnant pool.

The population in the locality of this epidemic live in small, badly constructed and poorly ventilated cabins, situated on the low grounds, near the water courses, but sometimes on the hill sides, and often partially buried in the ground, surrounded closely by the growing vegetation. The floors of their houses are composed of planks or slabs loosely laid on timbers very often completely buried in the earth, though frequently over a rude cellar of various dimensions, wherein is stored away cabbage, turnips, potatoes and other vegetables, for the convenience of obtaining them in the cold of winter. These reservoirs always contain a portion of vegetables left over the winter's use, which here remain until the cellar is wanted the following season.

The loose condition of the floor offers a free passage for dish water and other slops, which are daily poured into this receptacle; and once a week, on washing day, the floor is flooded with soap-suds most profusely. Thus from the amount of water thrown into these cellars or pits beneath the floor, they become partially filled, and the unusual and protracted heat of this summer, acting upon the moisture and vegetable matters, rendered these sinks the hotbed of malaria and it is believed a fruitful source of disease. To this may be added the additional fact, that the numerous streams in this particular section of country, of ordinary seasons furnishing pure running water, were so dried up by the long continued and extreme heat of summer, that the accumulated filth and vegetable matters washed from the adjacent hills and usually covered by water, and consequently harmless, were exposed to the direct action of the sun's rays, thus furnishing all the essentials for the abundant generation of malaria.

Dysentery made its appearance in the lowest and dampest grounds, near the small streams, and was observed to prevail more extensively, and with greater severity, where we know the causes of malaria were abundant, and to which we are disposed to attribute, in great part its origin. Those persons who were fed best, clothed best, and who had the best water, and were most cleanly as to person and habitation, were the ones who generally escaped the violent form of the disorder, although surrounded on all sides by it. When the disease occurred on the hills around, immediately in the vicinity of the most malignant cases, it was, as heretofore stated, much milder, and more easily managed.

The inhabitants are poor and are not always provided with a sufficiency of wholesome food, and as a consequence partake freely, at this season of the year, of green corn and other crude vegetables, to the exclusion of a more nutritious and healthy diet.

Are not the causes which we have thus briefly enumerated sufficient to justify us in the conclusion, that although this particular section of country is generally exempt from diseases of malarial origin, that this epidemic owed its existence, mainly, to this cause? To the above causes may we not add that of a general epidemic tendency, which doubtless existed throughout Northern Kentucky?

The initiatory symptoms of dysentery, as manifested in the epidemic of which I write, were by no means uniform. In some cases the first symptom was severe griping pains in the lower bowels, with an irresistible tenesmus, with very frequent but small discharges of mucus, slightly stained with blood. In this class of cases, fever was one of the first and most marked of the symptoms, with the usual train of phenomena accompanying it, headache, quick, tolerably hard, but small pulse, loss of appetite, thirst, skin not very hot, but dry and husky, tongue dry and covered with a short brownish fur, with red tip and edges, countenance expressive of intense anxiety, features sharp great tenderness extending over the region of the stomach as well as the entire abdomen. Cases thus violently ushered in, without premonitory symptoms, rapidly ran from bad to worse, every symptom becoming more severe, especially the tormina, which at times was almost insupportable; the desire to stool incessant, with inability to pass anything but the smallest quantity of mucus, and this attended with no relief. Very soon, if the disease was not arrested, the discharges, though not so frequent, became muco-purulent, the pulse very small, tongue glazed, sometimes cracked, the fever assumed a low typhoid character, with decided tendency to collapse—a feature noticeable in nearly all the more violent attacks, and always a most formidable symptom.

In another class of cases, the first symptom which attracted the attention of the patient was pretty free discharges of blood with fæcal matter, accompanied to a considerable extent by griping pains in the lower bowels, at first not very severe, but if not speedily met by appropriate medication, hastily running into a serious form of the disease. The discharges, which at first were not very frequent, and which were followed by a temporary remission of the characteristic pain, became more frequent and the tormina and tenesmus more severe.

Fever, which had not previously been very prominent, now sets in with all the symptoms usually accompanying the febrile movement. The discharges, now more frequent, become quite small, consisting of blood and mucus with occasionally a little fæcal matter, with white shreds, seemingly of false membrane, and flakes of vitiated mucus and bran-like scales.

In the early stage of this form of attack, the pulse is generally full with some hardness, the tongue covered with a rather short, yellow fur, with redness of the tip and edges.

In quite a number of cases, the symptoms of an ordinary diarrhœa, continuing for a day or two, unattended by fever, but occasionally some griping pains in the bowels, were the first to attract attention. Such cases, if not promptly met, soon assumed the peculiar type of the epidemic, and when fairly developed were as troublesome to the physician, and as torturing to the patient as those more violent in the onset. Suppression of urine was a very constant symptom in this, as well as in most of the other forms of the disease.

Treatment.—In giving the treatment I shall confine myself to the principles and plan of treatment generally pursued in the epidemic which I have here attempted to sketch. Dysentery, being an inflammatory disease, requires depletion in proportion to the violence of the attack, the strength, habits and age of the patient, and the prevailing tendency at the time to assume a sthenic or asthenic character. During some seasons, and in some epidemics, the prevailing tendency is to sink rapidly into a typhoid state; at others, diseases of almost every kind run their course without this tendency to any marked extent—they are active—sthenic in character, and bear depletion well, in fact cannot be successsfully managed, in many instances, without its most active employment. At other times, diseases are ushered in bearing marks of being highly inflammatory—requiring apparently the most active treatment—but from the tendency to assume a typhoid character, we must be very cautious in the employment of depletory remedies, or before we are aware of it, our patient slips through our hands. Hence, from the decided adynamic tendency of this epidemic, one of the most important remedies in acute disease, viz.—general bleeding, was rendered unavailable, unless the patient was seen very early after the attack, and then only in a very limited number of cases was it deemed advisable to resort to it.

Another form of depletion and the one upon which we mainly depended, was in the use of laxatives, or rather gentle purgatives, from which we received decided benefit. When called to a patient, if general bleeding was not admissible, we usually gave small doses of calomel and ipecac combined with Dover's powder, every few hours, with a view of exciting the secretions. This was followed, in six or eight hours, by sulphate of magnesia or castor oil, with a few drops of laudanum to prevent griping. Having cleared the bowels of their vitiated secretions, and procured bilious evacuations, all forms of mercury were withdrawn and the bowels quieted by decided doses of Dover's powder and sulphate of morphia, repeated as indicated.

If there was much fever, as manifested by thirst, dryness of the skin, &c., ipecac was added to the anodyne. As an adjunct to the anodyne, in allaying pain, we have found warm fomentations assiduously applied to the abdomen of the greatest benefit. As to laxatives in cases evidently typhoid in tendency, we prefer castor oil and spirits of turpentine combined, with the addition of the usual anodyne, to the sulphate of magnesia, as being better suited to the in lications to be fulfilled.

When the disease is pretty strongly marked as inflammatory, we prefer the saline purgatives, as depleting more rapidly, and as has been said, relieving the local congestion directly. The sulphate of magnesia and castor oil may be rendered more bland and less irritating, and their effiiciency not diminished, by the addition of a small portion of molasses or mucilage of gum arabic to each dose.

The warm bath we consider one of the most valuable remedies in the treatment of the disease, and when practicable we never fail to employ it, and with the best results. It relieves pain, allays thirst, and by cleansing and relaxing the skin, promotes perspiration.

A warm bath at bed time, with a Dover's powder and a cup of warm tea, seldom fails to produce diaphoresis.

Blisters were but little used, from the fact that we derived but little benefit from them, over and above that derived from sinapisms applied over the abdomen and stomach until the surface was almost vesicated, keeping up the effect of the mustard by the use of rubefacient applications, as aqua ammoniæ, spirits of turpentine, spirits of camphor, &c. From the proper use of the above, the sufferings of our patients were generally greatly abridged.

Emetics were not employed, for two reasons—first, because of the presence of gastric symptoms which we believed would not justify their use, and secondly, the most fatal form of the disease—viz: collapse—was preceded by active vomiting, accompanied by rapid diminution of temperature, which we never succeeded in restoring, though varied plans of treatment were adopted—all were alike unsuccessful. The collapse in this disease we consider as more fatal, if possible, than that of cholera itself.

Injections were used, but no decided benefit seemed to follow their administration.

In our course of treatment we gave mercury occasionally, in small doses, combined with the anodyne, sufficient to keep up the biliary secretion, and no more. We moved the bowels once in the twenty-four hours, for the purpose of ridding them of the acrid secretions they contained, then procure quiet by the use of decided anodynes, as before.

Mercury was found not to be good treatment in the malignant form of the disorder.

Last summer and autumn, in the neighborhood of the famous Blue Lick Springs, dysentery prevailed to a very considerable extent, and was very successfully treated with Blue Lick water and Dover's powder alone. The disease did not occur in a malignant epidemic form, but cases of very considerable violence rapidly recovered under the use of the remedies above named.

These remedies were so successful in the past summer as to warrant their further employment. If opportunity should again present itself, I would feel myself justified in a further use of them.

